# The impact of health information technology on disparity of process of care

**DOI:** 10.1186/s12939-015-0161-3

**Published:** 2015-04-01

**Authors:** Jinhyung Lee

**Affiliations:** Department of Economics, Sungkyunkwan University, Seoul, Korea

**Keywords:** Health information technology, Disparity, Process of care, Random intercept and slope model

## Abstract

**Introduction:**

Disparities in the quality of health care and treatment among racial or ethnic groups can result from unequal access to medical care, disparate treatments for similar severities of symptoms, and wide divergence in general health status among individuals. Such disparities may be eliminated through better use of health information technology (IT). Investment in health IT could foster better coordinated care, improve guideline compliance, and reduce the likelihood of redundant testing, thereby encouraging more equitable treatment for underprivileged populations. However, there is little research exploring the impact of health IT investment on disparities of process of care.

**Methodology:**

This study examines the impact of health IT investment on waiting times – from admission to the date of first principle procedure – among different racial and ethnic groups, using patient and hospital data for the state of California collected from 2001 to 2007. The final sample includes 14,056,930 patients admitted with medical diseases to 316 unique, acute-care hospitals over a seven-year period. The linear random intercept and slope model was employed to examine the impacts of health IT investment on waiting time, while controlling for patient, disease, and hospital characteristics.

**Results:**

Greater health IT investment was associated with shorter waiting times, and the reduction in waiting times was greater for non-White than for White patients. This indicates that minority populations could benefit from health IT investment with regard to process of care.

**Conclusion:**

Investments in health IT may reduce disparities in process of care.

**Electronic supplementary material:**

The online version of this article (doi:10.1186/s12939-015-0161-3) contains supplementary material, which is available to authorized users.

## Background

Racial and ethnic disparities in health status and quality of health care often result from complex decisions made by providers, utilization managers, health system administrators, and other personnel within the health care system [1,2]. Some of these factors contribute to higher levels of discrimination toward certain groups – a situation that either directly or indirectly contributes to health-related disparities. Over the past two decades, racial and ethnic factors have been associated with unequal access to medical care, differing treatments for similar severity of symptoms, and general health status in the United States [1,3,4].

Nationwide programs aiming to promote health and prevent disease, such as Healthy People and Racial and Ethnic Approaches to Community Health (REACH 2010), focus on the elimination of racial and ethnic disparities in health care and treatment. These inequalities have been found to be important determinants of health disparities and differences in access to health care, preventive health services, positive treatment outcomes, and other inequalities. To eliminate racial and ethnic disparities in health, it is critical to understand the root of the problem.

Previous studies have demonstrated disparities in process of care. Some show that African Americans with diabetes are less likely to have undergone recommended process of care measures, such as glycated hemoglobin (A1C) and lipid measurements, than White [5,6]. Other studies have found disparities in the process of transplant wait-listing. Arce et al. [7] found that Black and Hispanic patients had significantly longer wait times than White patients from the start of dialysis to kidney transplant wait-listing. These studies, however, failed to identify ways to reduce disparities. The consistency of health care inequality is also problematic, as minority populations are projected to represent nearly half of the total U.S. population by the year 2050. The populations of the two largest minority groups (Hispanics and non-Hispanic Blacks) are projected to increase to a total of nearly 79 million by 2050 –70% and 300% increases, respectively [8].

Health information technology (IT) is a potentially significant avenue to correct this disparity. The National Health IT (NHIT) Collaborative for the Underserved was recently launched to address health disparities and promote optimal health through the effective use of health IT. Tools such as patient electronic medical records (EMRs) and computerized physician order entry (CPOE) are vital for achieving health care reform due to their potential to increase access to health care, improve care delivery systems, reduce medical errors, and improve efficiency and quality of care [9-12]. The United States government has recognized the role of health IT to reduce health care costs and prevent medical mistakes, and is increasing investment in this area as a central part of health reform. The Health Information Technology for Economic and Clinical Health (HITECH) Act, which was signed into law in 2009 in order to promote the adoption and meaningful use of health IT, allocated nearly $27 billion to subsidize health IT adoption, potentially investing $2-$10 million per hospital. The growing interest and investment in health IT is based on the goals of cost and quality control: the adoption of health IT is considered a means of containing costs and improving the efficiency and quality of health care.

Health IT may eliminate health care disparities [1]. For example, the use of a clinical decision support system (CDSS) within an EMR system could prompt physicians to give evidence-based recommendations when reporting diagnostic and screening tests, as well as to provide immunizations for primary prevention and chronic disease management. Health IT would also foster better coordinated care (thereby increasing processing speed and reducing the likelihood of redundant testing [13]) in order to encourage equitable treatment for minority populations by eliminating any potential biases that the provider might have in terms of clinical judgment [14]. Improvements in care are also possible when health IT-based treatment detects factors that increase the risk of a particular group [12].

Few studies have examined the impact of health IT on racial or ethnic disparities in access to and quality of health care. The existing literature focuses on health IT adoption, lacking analyses of the relationships between health IT adoption and racial or ethnic disparities in outcomes or process of care [9,15,16]. In order to address this gap, in this paper we examine the effects of health IT on the process of care, focusing on the waiting time from a patient’s date of admission to the date of the principle procedure.

We examine two hypotheses. First, health IT investment reduces waiting time from the patient’s date of admission to the date of the principle procedure among medical patients, after controlling for important patient and hospital characteristics. Health IT may reduce the patient’s waiting time by facilitating cross-provider communication, helping hospital staff to manage clinical information, fostering better coordination of care, and increasing processing speed [13]. Second, waiting time reductions associated with health IT investment are more pronounced among minorities. According to the Institute of Medicine (2003), biases, prejudices, and negative racial stereotypes are potential sources of disparity. Thus, health IT may reduce waiting time disparities by preventing negative racial stereotyping through improved guideline compliance.

We examined patient and hospital data from the California Office of Statewide Health Planning and Development (OSHPD) collected from 2001 to 2007 in order to test these two hypotheses.

## Methods

### Data

The OSHPD data are composed of hospital and patient information. The hospital-level OSHPD data provide hospital characteristics such as ownership type, number of beds, system affiliation, and teaching status. The OSHPD is noteworthy for its inclusion of information on health IT expenditures and depreciation, which we used to construct measures of health IT investment [17]. This IT-investment measure represents the amount of money any given hospital may allocate to IT, although these figures may not represent real IT use in hospitals. Patient-level OSHPD data provides patient characteristics, total cost of care, length of stay, admission type, payment source, patient disposition, and disease characteristics for all hospitalized patients.

### Inclusion and exclusion criteria

We included only patients with routine discharges – defined as patients scheduled for follow-up care at a physician’s office or those discharged under hospice care – and excluded patients referred to acute care, the emergency department, nursing home, intermediate care, or home health services. We also excluded patients transferred to the hospital from another facility. Patients transferred from facilities near high-quality hospitals tend to have more severe medical conditions, and if investment in health IT is correlated with unobserved patient severity, estimations of the effect of health IT investment on process of care would be biased [18]. We focused on medical diagnosis-related groups (DRG) because medical disease is less complicated than surgical disease and may be easily captured by health IT. In addition, if surgery is the principle procedure, the likelihood that its medical necessity is related to non-medical factors such as race or ethnicity may be lower, as time is a preferred factor. Thus, all encounters in which surgery was the principle procedure were excluded from the analysis. The principle procedures are coded according to the ICD-9-CM and include more than 1,200 procedure codes including left heart cardiac catheterization (ICD-9-CM: 3722), hemodialysis (ICD-9-CM: 3995), and colonoscopy (ICD-9-CM: 4523).

A hierarchical data set with two levels was employed in this study: the first level is admission and the second level is hospitals. The panel data represented only the second level of data (hospital); therefore, we were only able to follow hospitals over the sample period. The unit of analysis is admission. The final sample includes 14,056,930 admissions in 316 unique, acute-care hospitals in the state of California over a period of seven years.

### Dependent variables

The dependent variable is defined as the number of days between the patient’s date of admission and date of the principle procedure. The California OSHPD began providing this information in 2001. As the key explanatory variable, health IT investment was measured as a dollar investment in both capital and labor as related to IT. Health IT capital included hardware and software, while health IT labor included salaries, wages, and benefits related to IT. The OSHPD data place all IT expenditures within the “data processing” section of the hospital’s financial statements. Health IT investments were extracted from each hospital’s balance sheet [17].

### Independent variables

As the independent variables, we controlled for patient and disease characteristics, including age (1-17, 18-34, 35-64 and 65 years of age and older), gender, race (White and non-White, including Black, Asian/Pacific Islander, Native American/Eskimo/Aleut and others), and diagnosis-related group (DRG) or DRG weight (a measure of the usual amount of inpatient resources consumed by a patient of that type). The models included five categories describing payment source: 1) Medicare; 2) Medical; 3) private (payment covered by private, non-profit, or commercial health plans); 4) self-insurance (payment directly by the patient, personal guarantor, relatives, or friends); and 5) other (workers’ compensation, indigent programs, other government programs, and any third-party payments not included above). We also controlled for hospital characteristics, including ownership (profit, not-for-profit, or government), system member, teaching status, bed size, and rural location.

### Analysis

We utilized the Hauseman test to check the effects of within and between at the patient-admission (first level) and hospitals (second level), and found that the random-effects model is more appropriate than the fixed-effect model (*X*^2^ = 234.88, P-value < 0.001). The null hypothesis is that differences in coefficients are not systematic. Thus, the linear random effects model was applied with random intercept and slope. The random intercept and slope model had a lower value of log likelihood (-1514657.7) than the random intercept model (-1515191.7). In addition, we tested a serial auto-correlation of panel data [19] and found that the null hypothesis (no first-order auto-correlation) could not be rejected (p-value = 0.44).

The dependent and independent variables were taken as natural logs because they were skewed. The mean, standard deviation, skewness, and kurtosis of waiting time were 2.845, 3.305, 13.4 and 964.2, respectively. Also, to control for outliers, any residuals larger or smaller than three standard deviations from the mean were eliminated. Lastly, we calculated the multi-collinearity between independent variables and found the variance inflation factors (VIF) of all variables to be less than 4, indicating that multi-collinearity was not a problem [20].

Thus, the relationship between health IT investment and waiting time from admission to the date of the principle procedure was evaluated using a linear random intercept and coefficient model. This model also accounted for the clustering of admissions within hospitals in order to control for within-hospital correlation and for patient and disease characteristics, payment source, and hospital characteristics. Analyses were performed using STATA version 11.2.

## Results

Table 1 describes the characteristics of patients and hospitals. Persons 65 years of age and over accounted for the majority of patients (50.8%), and their waiting time was the longest (2.978 days) among all age groups. Males accounted for 45.9% of patients, with a waiting time slightly shorter than that of female patients. Patients with medical insurance accounted for only 19.5% of the total; however, their waiting times were the longest. Non-White patients accounted for 37.9% of the total, with an average waiting time three or more hours longer than that of White patients. There were also variations in waiting times from admission to principle procedure that were related to racial and group characteristics and that spanned across hospital characteristics. Not-for-profit, network, non-teaching, and rural hospitals had shorter waiting periods than those of their counterparts (all p < 0.01). The average DRG weight was 1.18. The average number of beds was 339 and average health IT investment was $14.4 million.

**Table 1:** **Wait times to first major procedure according to patient and hospital characteristics (sample size: 14,056,930)**

**Variable**		**Percent**	**Mean** ^**1**^	**(SD)**	**P-Value**
Age (years)					P < 0.01
	1-17	1.4%	2.553	(3.344)	
	18-34	7.3%	2.555	(3.405)	
	35-64	40.5%	2.740	(3.397)	
	65 and older	50.8%	2.978	(3.207)	
Sex					P < 0.01
	Male	45.9%	2.827	(3.105)	
	Female	54.1%	2.845	(3.417)	
Payment Source					P < 0.01
	Medicare	51.3%	2.982	(3.162)	
	Medical^2^	19.5%	3.111	(4.168)	
	Private	19.9%	2.367	(2.671)	
	Self	4.1%	2.479	(2.970)	
	Other^3^	5.3%	2.607	(3.202)	
Race					P < 0.01
	White	62.1%	2.796	(3.136)	
	Non-White	37.9%	2.925	(3.563)	
					P < 0.01
Ownership					P < 0.01
	Profit	16.5%	2.918	(3.162)	
	Not-for-profit	65.3%	2.823	(3.252)	
	Government	18.1%	2.858	(3.604)	
System					P < 0.01
	Non-system		2.901	(3.516)	
	System	55.8%	2.800	(3.127)	
Teaching hospitals					P < 0.01
	Non-teaching	77.5%	2.811	(3.124)	
	Teaching	22.5%	2.968	(3.897)	
Location					P < 0.01
	Non-Rural	95.6%	2.861	(3.348)	
	Rural	4.4%	2.528	(2.448)	
DRG weight			1.18	(0.664)	
Number of beds			339	(199)	
Health IT			$14.4 m	(20.1 m)	

^1^Number of days between the patient’s date of admission and the date of the principle procedure.

^2^Medicaid is known as Medical in California.

^3^Other category includes Race: Native American/Eskimo/Aleut; Payment Source: workers’ compensation, indigent programs, other government and any third party payment not included above.

### The process of care

Hypothesis 1: Health IT investment reduces waiting time from a patient’s date of admission to the date of the principle procedure, after controlling for important patient and hospital characteristics.

We examined the relationship between IT investment and waiting time from admission to the principle procedure. The regression results showed that health IT investment was associated with reduced waiting time after controlling for patient characteristics, disease characteristics, payment source, and hospital characteristics (Table 2). This implies that a 10% increase in health IT investment is associated with a 0.9% waiting time reduction.

**Table 2:** **Regression results for medical DRGs (unit of analysis: admission)**

**Variables**		**Coefficient**
		**(std. err)**
Age (years)	Ref (1-17)	
	18 to 34	−0.007
		(0.006)
	35 to 64	0.050***
		(0.006)
	65 and older	0.094***
		(0.006)
Sex	Ref (Female)	
	Male	−0.019***
		(0.001)
Payment Source	Ref (Medicare)	
	Medical^**1**^	0.027***
		(0.002)
	Private	−0.116***
		(0.002)
	Self	−0.107***
		(0.004)
	Other	−0.046***
		(0.003)
DRG weight		0.173***
		(0.001)
Health IT		−0.009***
		(0.002)
Race	Non-White	0.037*
		(0.021)
Non-White × Health IT		−0.002*
		(0.001)
Ownership	Ref (Profit)	
	Not-for-profit	−0.041***
		(0.009)
	Government	−0.050***
		(0.013)
Teaching status		−0.025
		(0.018)
Network hospital		−0.004
		(0.010)
Licensed beds		0.0001***
		(0.000)
Rural hospital		−0.088***
		(0.014)
Constant		0.625***
		(0.031)

***p < 0.01, **p < 0.05, *p < 0.1, ^**1**^Medicaid is known as MediCal in California.

This regression examined the effect of IT investment on waiting time after controlling for other independent variables.

We used linear random effects model because number of days is not count data, but duration data which means occurrences of the counted behavior are not independent of each other. Also, other studies used linear model to estimate the number of days [21-23]. However, we tried multilevel Poisson regression and presented regression results in Additional file 1. The results are similar to linear regression model in Table 2 even though the coefficient of health IT investment was not significant and that of interaction term of IT investment and white was a little bit larger in amount.

### The process of care across races/ethnicities

Hypothesis 2: Reductions in waiting time associated with health IT investment are more pronounced among minorities.

Non-White patients had waiting times at least three hours longer than that of Whites (Table 1). Health IT investment was associated with shorter waiting times, as shown in the results under Hypothesis 1. Therefore, the question becomes: Can health IT investment further reduce non-White waiting times in order to create racial parity? To examine this, we investigated how IT investment was related to waiting times among different races and ethnicities. As expected, the non-White patient groups had longer waiting times than the White patient group (P < 0.001). In addition, health IT investment was associated with shorter waiting times for non-Whites. A 10% increase in health IT investment is associated with 0.4% waiting time reduction in non-Whites (Table 2).

Other factors also associated with shorter waiting times in Table 2 were patient characteristics including age (children aged 1 to 17 years), gender (male), and payment source (including private, self, and other health insurance). Hospital characteristics were also associated with waiting time. Across ownership, not-for-profit and government-sponsored hospitals had shorter waiting times than for-profit hospitals. Bed size also had an effect, although the effect was small (coefficient = 0.0001). Rural hospital status was also associated with shorter waiting times; however teaching and system-member hospital statuses were not associated with waiting time.

### Robustness check

In order to produce valid results, unobserved changes in hospital quality should not be correlated with IT investment [24]: health IT investment would appear to decrease quality if invested in response to problems related to quality. For example, in this study, it is possible that the differential changes (increases or reductions) in wait times led to increased investment, rather than vice versa. Such reverse causality would weaken our main findings with regard to the impacts of health IT on waiting time. Therefore, we examined the causal relationship between IT investment and waiting time. In order to test this potential identification problem, we estimated the models using one-year preceding (t + 1) and lagged (t-1) IT investment (Table 3), and found that future IT investment is not correlated with current waiting time, while lagged IT investment continues to reduce waiting time, indicating that reductions in wait times do not lead to high IT investment.

**Table 3:** **Regression results for medical DRGs with one year preceding and lagged IT investment (unit of analysis: admission)**

**Variables**		**Coefficient**	**Coefficient**
		**(std. err)**	**(std. err)**
Age (years)	Ref (1-17)		
	18 to 34	−0.011	−0.006
		(0.007)	(0.0060
	35 to 64	0.049***	0.051***
		(0.006)	(0.006)
	65 and older	0.092***	0.094***
		(0.007)	(0.006)
Sex	Ref (Female)		
	Male	−0.021***	−0.018***
		(0.001)	(0.001)
Payment source	Ref (Medicare)		
	Medical^**1**^	0.028***	0.028***
		(0.002)	(0.002)
	Private	−0.117***	−0.116***
		(0.002)	(0.002)
	Self	−0.107***	−0.10***7
		(0.004)	(0.004)
	Other	−0.045***	−0.046***
		(0.004)	(0.003)
DRG weight		0.183***	0.173***
		(0.001)	(0.001)
Health IT (t + 1)		−0.003	
		(0.002)	
Health IT (t-1)			−0.004***
			(0.002)
Race	Non-White	0.000	0.001
		(0.002)	(0.002)
Ownership	Ref (Profit)		
	Not-for-profit	−0.062***	−0.045***
		(0.010)	(0.010)
	Government	−0.068***	−0.050***
		(0.014)	(0.013)
Teaching status		−0.024	−0.029
		(0.019)	(0.018)
Network hospital		−0.008	−0.004
		(0.011)	(0.010)
Licensed beds		0.0001***	0.0001***
		(0.000)	(0.000)
Rural hospital		−0.085***	−0.084***
		(0.015)	(0.014)
Constant		0.535***	0.547***
		(0.031)	(0.027)

***p < 0.01, **p < 0.05, *p < 0.1, ^**1**^Medicaid is known as MediCal in California.

This regression examined the effect of IT investment on waiting time after controlling for other independent variables.

## Discussion

We detected some degree of disparity in waiting time from the patient’s date of admission to the date of the principle procedure across races and ethnicities. The average waiting time among White patients was significantly shorter than that of non-Whites. This type of bias could be reduced by increasing health IT investment, as such investments improve guideline compliance, support better health care decision-making and better coordinated care, increase processing speed, and reduce the likelihood of redundant testing. We found that health IT investment was associated with shorter waiting time and therefore potentially eliminates health care disparities in process of care. Waiting time disparities among races and ethnicities may be attributed to biased relationships between patients and physicians. If health IT improves guideline compliance, clinician decisions will become more consistent and objective. We found that waiting time reductions associated with health IT investment were slightly more pronounced among minorities. This implies that minorities could benefit from health IT in terms of process of care, and is consistent with previous results. For example, Tucker and Miller [12] investigated the impact of EMRs on infant and neonatal mortality rates among both White and African American babies, concluding that EMR adoption was significantly associated with a reduction in infant mortality rate, and that the benefits were greater for African Americans [12].

Moreover, interestingly, we found disparities in waiting time across gender and payment sources (Table 2). Males were the most advantaged group in process of care, with shorter waiting times than females. Patients with private insurance also had shorter waiting times than those with public insurance, including Medicare and Medicaid. Those with private insurance were the most advantaged group. Patients with Medical (as Medicaid is known in California) insurance had much longer waiting times than those with other forms of insurance. Patients with Medical insurance had lower incomes, appeared to have fewer choices with regard to health care, and were also the most disadvantaged group with regard to health care. If care is delayed due to insurance status and delayed care is associated with health care outcomes, the result will be more serious health care outcomes.

The differing effects of health IT on waiting times across hospital characteristics are shown in Table 2. Hospital ownership status played a significant role in this area: health IT investment had a greater impact on waiting time reduction in not-for-profit hospitals than in for-profit and government-sponsored hospitals. However, health IT investment led to longer waiting times in teaching hospitals. We also found that large and non-rural hospitals had greater waiting time reductions due to health IT investment than did their counterparts, which implies that large and non-rural hospitals have more infrastructure and therefore would gain the greatest benefit from the use of health IT.

We examined possible reverse causality, as differential changes in wait times may lead to increased health IT investment. This type of reverse causality would invalidate the main findings of the study. However, reduced waiting times do not lead to high IT investment, indicating that there is no reverse causality between waiting time and health IT investment and that confirm the main findings.

This study is subject to several limitations. First, specific providers may play significant roles in creating disparities in process of care. However, physician data were not available. If disparities in wait times across races and ethnicities resulted from clinician behavior, the coefficients may be biased. Second, we only focused on medical diseases. While waiting time disparities in this area are less complicated than in other areas and may be easily captured by health IT, those related to surgical disease may not be as easily captured. Medical necessity for surgical disease may be less related to non-medical factors such as race, since time is a more important factor. Third, there may be factors confounding the relationship between IT investment and wait times. For example, differences in quality, efficiency and patient-centered care associated with IT investment may explain the observed relationship between IT and wait times. Fourth, the IT investment measured in this study was a proxy for IT use. Thus, this measure may not represent real IT use in hospitals. In addition, IT investment included various components, some of which may be used for business management systems such as financial, human resource, and material management systems. Thus, a business-related IT system may be less likely to impact patient waiting time, unless the procedure was pre-approved by a payer for hospital reimbursement.

## Conclusion

In this study, we found that health IT investment is associated with shorter waiting time from the patient’s date of admission to the date of the principle procedure. We also determined that waiting time reductions associated with health IT investment were more pronounced in non-White patients than in White patients. These results suggest that health IT investment could reduce disparities in process of care, although this area requires further study.

## Additional file

Additional file 1:
**Poisson regression results for medical DRGs.**
