# Supplementary material for: The impact of health information technology on disparity of process of care
Source: Int J Equity Health. 2015 Apr 1;14:34. doi: 10.1186/s12939-015-0161-3 (PMC4392633; doi:10.1186/s12939-015-0161-3)
Supplement: Additional file 1: — Poisson regression results for medical DRGs. [file 12939_2015_161_MOESM1_ESM.docx]

Appendix Table 1 Poisson regression results for medical DRGs

(Unit of analysis: admission)

| Variables | | Coefficient |
| --- | --- | --- |
|  |  | (std. err) |
| Age (years) | Ref (1-17) |  |
|  | 18 to 34 | −0.030 |
|  |  | (0.053) |
|  | 35 to 64 | 0.021 |
|  |  | (0.058) |
|  | 65 and older | 0.059 |
|  |  | (0.060) |
| Sex | Ref (Female) |  |
|  | Male | −0.004 |
|  |  | (0.005) |
| Payment source | Ref (Medicare) |  |
|  | Medical^1^ | 0.062*** |
|  |  | (0.011) |
|  | Private | −0.175*** |
|  |  | (0.010) |
|  | Self | −0.127*** |
|  |  | (0.020) |
|  | Other | −0.074*** |
|  |  | (0.023) |
| DRG weight |  | 0.204*** |
|  |  | (0.006) |
| Health IT |  | −0.016 |
|  |  | (0.011) |
| Race | Non-White | 0.230** |
|  |  | (0.115) |
| Non-White*Health IT |  | −0.013* |
|  |  | (0.007) |
| Ownership | Ref (Profit) |  |
|  | Not-for-profit | −0.068** |
|  |  | (0.028) |
|  | Government | −0.085** |
|  |  | (0.040) |
| Teaching status | | −0.013 |
|  |  | (0.048) |
| Network hospital | | −0.036 |
|  |  | (0.022) |
| Licensed beds | | 0.0003*** |
|  |  | (0.0001) |
| Rural hospital | | −0.056 |
|  |  | (0.038) |
| Constant |  | 1.045*** |
|  |  | (0.167) |

*** p < 0.01, ** p < 0.05, * p < 0.1, ^1^Medicaid is known as MediCal in California.

This regression examined the effect of IT investment on waiting time after controlling for other independent variables.
